# Drinking Water Fluoride Levels for a City in Northern Mexico (Durango) Determined Using a Direct Electrochemical Method and Their Potential Effects on Oral Health

**DOI:** 10.1155/2013/186392

**Published:** 2013-11-20

**Authors:** Nelly Molina Frechero, Leonor Sánchez Pérez, Enrique Castañeda Castaneira, Anastasio Oropeza Oropeza, Enrique Gaona, José Salas Pacheco, Ronell Bologna Molina

**Affiliations:** ^1^Health Care Department, Autonomous Metropolitan University (UAM), Xochimilco, 04960 Mexico City, DF, Mexico; ^2^Scientific Research Institute, Juárez University of the Durango State (UJED), 34000 Durango, DGO, Mexico; ^3^Oral Pathology, School of Dentistry, Juárez University of the Durango State (UJED), 34000 Durango, DGO, Mexico

## Abstract

Fluoride is ingested primarily through consuming drinking water. When drinking water contains fluoride concentrations >0.7 parts per million (ppm), consuming such water can be toxic to the human body; this toxicity is called “fluorosis.” Therefore, it is critical to determine the fluoride concentrations in drinking water. The objective of this study was to determine the fluoride concentration in the drinking water of the city of Durango. The wells that supply the drinking water distribution system for the city of Durango were studied. One hundred eighty-nine (189) water samples were analyzed, and the fluoride concentration in each sample was quantified as established by the law NMX-AA-077-SCFI-2001. The fluoride concentrations in such samples varied between 2.22 and 7.23 ppm with a 4.313 ± 1.318 ppm mean concentration. The highest values were observed in the northern area of the city, with a 5.001 ± 2.669 ppm mean value. The samples produced values that exceeded the national standard for fluoride in drinking water. Chronic exposure to fluoride at such concentrations produces harmful health effects, the first sign of which is dental fluorosis. Therefore, it is essential that the government authorities implement water defluoridation programs and take preventative measures to reduce the ingestion of this toxic halogen.

## 1. Introduction

Water is a scarce resource in Mexico; thus, a plan was developed to exploit the country's underground aquifers. Because fluoride is abundant in the earth's crust, it is a common drinking water contaminant, and high fluoride concentrations in contaminated drinking water are rapidly becoming an endemic public health problem. The drinking water fluoride concentration limit set by the World Health Organization (WHO) is 1 part per million (ppm), but this limit depends on an area's geological characteristics. For Mexico, the drinking water fluoride limit is 0.7 ppm, as described by the national regulatory standard NOM-013-SSA2-2006 [1, 2]. Fluoride is an abundant ion in the earth's crust that is highly electronegative; thus, it combines with various soil substances to form fluoride salts. When water passes through the soil, these fluoride compounds dissolve and increase the fluoride groundwater concentration in the presence of lithium, cesium, chloride, and bromide [3]. The groundwater composition is primarily determined by its time in the aquifer and the materials and ions dissolved therein. To establish suitable groundwater for human consumption, one must consider the water's hardness and the concentrations of salts, iron, carbon dioxide, sulfur compounds, fluoride, arsenic, lead, chromium, manganese, and other ions [4].

The most common fluoride minerals in the earth's crust are fluorspars (fluorite, cryolite, and apatite), which are typically composed of calcium, fluoride, carbonates, and sulfates [5]. Fluoride is an extremely reactive element that combines with other elements through ionic or covalent bonds and is primarily found in igneous and alkaline rocks. Fluorite is the most abundant compound formed and is found in granite, gneiss, and pegmatite. The fluoride concentration in aquifers depends on various factors, including the fluoride concentrations of the surrounding minerals; the mineral decomposition, dissociation, and dissolution rates; the groundwater time in the aquifer; and the reaction kinetics. These factors determine the fluoride solubility in water for a given type of aquifer [6].

Fluoride contamination in drinking water can reach concentrations higher than 1 ppm, and fluoride can be ingested via bottled beverages, table salt, toothpaste, and various foods. Chronic ingestion and consumption of fluoridated products can cause diseases, such as dental and skeletal fluorosis, and increase the risk of developing kidney problems and cancer [2, 7]. Furthermore, consuming high fluoride concentrations reportedly impairs human mental development and lowers IQ in school-aged children [7, 8].

Dental fluorosis occurs during odontogenesis and tooth development and produces hypomineralization as well as surface and subsurface enamel porosity. Dental fluorosis can exhibit varying degrees of severity, which cause tooth color changes and generate serious aesthetic, functional, and physiological problems. Thus, fluorosis is a public health problem with high treatment costs [9]. 

In 1996, a list was developed of municipalities in five states in which iodized and fluoridated salt should not be distributed due to high fluoride concentrations in the drinking water, including the state of Durango [10, 11].

In 1997, Mexico reported that more than five million people were chronically exposed to high fluoride levels through drinking water [12]. Because exposure to high fluoride levels is a major public health problem, it is essential to assess the fluoride levels in the drinking water supply for the city of Durango, particularly because the city fluoride concentrations have not been studied, and children exhibit clinical dental fluorosis symptoms. Therefore, it is important to analyze the drinking water fluoride concentrations in this area to determine the contribution of this mineral to potential negative health outcomes caused by exposure to excessive fluoride levels.

The purpose of this study was to determine the drinking water fluoride concentrations for Durango, establish whether diseases related to different levels of fluoride exposure are present, and disseminate the results to prevent dental fluorosis, which is an early sign of high fluoride levels.

## 2. Materials and Methods

### 2.1. Study Area

**Figure 1:**
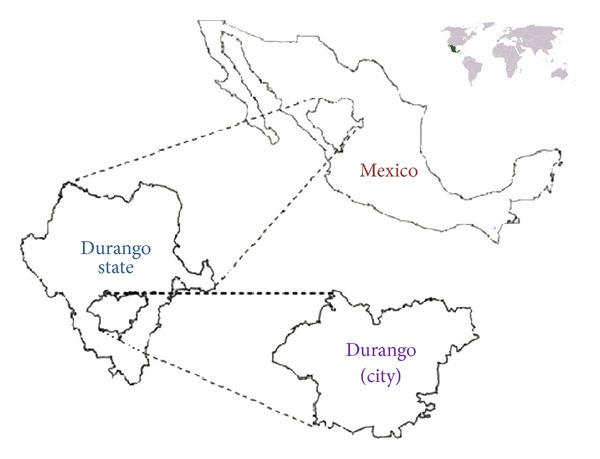
Map depicting the Durango city and state locations in the Mexican Republic.

The city of Durango (officially, the “Victoria de Durango”) is in Mexico and is the capital of the state of Durango (Figure 1). It is in the northern part of the country and is known by its inhabitants as “The Pearl of Guadiana” due to its location in the Valley of Guadiana, which is in the center of the state of Durango. The metropolitan region is 123,181 km^2^ with a population of 582,267, which represents more than one-third of the state's population [13]. The city is located in the far western end of the valley of Guadiana in northern Mexico and the central-western part of the Mexican high plains (*altiplano*). The average city elevation is 1880 meters above sea level (MASL). The city has only two notable geographic features: Mercado hill (2040 MASL) to the north of the city and Los Remedios hill (1980 MASL) to the west. According to the Köppen climate system, the city of Durango has a semitemperate climate, rain in the summer, and slightly colder weather in the winter with a few frosts and showers [14].

Durango's drinking water comes from the region's deep wells and aquifers; the population consumes groundwater extracted from the city's functioning wells. The first stage of this study included visits to the National Water Commission (CONAGUA, in Spanish) and Durango Municipal Water System to document the 66 wells distributed throughout the city, only 63 of which were functioning during the study period due to logistical defects. Durango's Municipal Water System operates the city's System of Wells, all of which are less than 250 meters deep.

**Figure 2:**
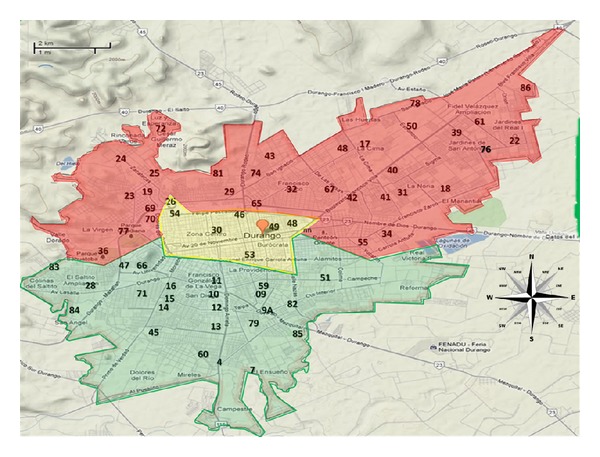
Location of wells in the city, which are distributed by geographic region (north, south, and central).

The 63 functioning wells were located throughout the northern, southern, and central sections of the city. Thus, the city was divided into three sections for this study, and the wells were enumerated and grouped by section (Figure 2).

In the second stage of the study, a method was developed to detect fluoride in the municipality drinking water. The water samples were collected over nine months (in a period of drought).

This was a descriptive and experimental study on drinking water fluoride concentrations.

### 2.2. Collection of Water Samples

Water samples were collected using 200 mL polyethylene bottles that were washed three times with deionized water; prior to collecting each sample, the bottles were labeled with the sample number, well, and location for identification.

The recommended method for fluoride analysis is the potentiometric method using an ion-selective electrode [15]. Herein, an Orion 4-star fluoride ion-selective electrode from the Thermo Electron Corporation was used. The potentiometric method facilitates fluoride detection in the concentration range 0.1 to 10 ppm; this method also requires a total ionic strength adjustment buffer solution (TISAB) to control pH, adjust the total ionic strength, and maintain the fluoride ion in a freely dissolved form in solution (Standard Methods 1998) [16]. The water samples were analyzed in the Fluorosis Laboratory at the Autonomous Metropolitan University in Xochimilco (UAM-X, in Spanish) on the day that they were collected.

A calibration curve was prepared using standard solutions with concentrations between 0.01 and 10 ppm. TISAB III was added to the standards to stabilize the ionic strength [16]. Fluoride readings for each sample were recorded using the Star Navigator and LabSpeed Navigator software packages. Data from triplicate measurements were analyzed using univariate statistical methods, and the means, standard deviations, and 95% confidence intervals were calculated for each sample; the mean concentration in each study area was calculated using the SPSS 21 software package.

## 3. Results

**Figure 3:**
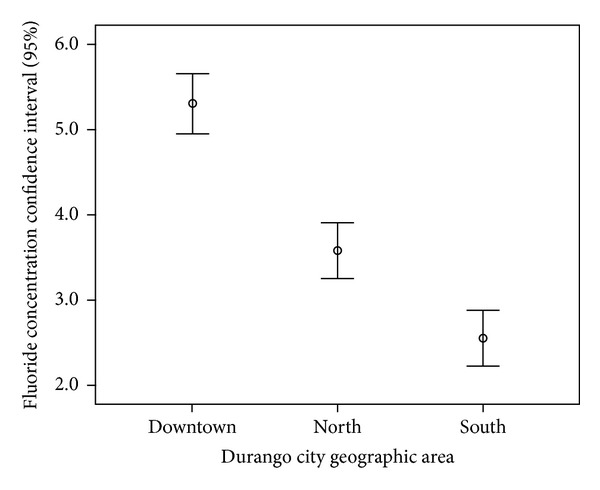
Fluoride concentration distribution in the city of Durango by region.

One hundred eighty-nine (189) water samples from 63 municipal wells were analyzed (the well water is the drinking water for Durango citizens), and the fluoride concentrations were between 2.22 and 7.23 ppm with a mean concentration of 4.313 ± 1.318 ppm (Figure 3). Ninety-three (93) samples from 31 wells in the northern region of the city were analyzed, and the fluoride concentrations ranged from 3.92 to 7.23 ppm with a mean of 5.30 ± 0.945. In the southern region, 75 samples from 25 wells were analyzed with a mean concentration of 3.5818 ± 0.786 ppm; in the center of the city, 21 samples from 7 wells were analyzed, and the fluoride concentrations ranged from 2.22 to 3.31 ppm with a mean of 2.5514 ± 0.363. These results are presented in Table 1 and Figure 3.

**Figure 4:**
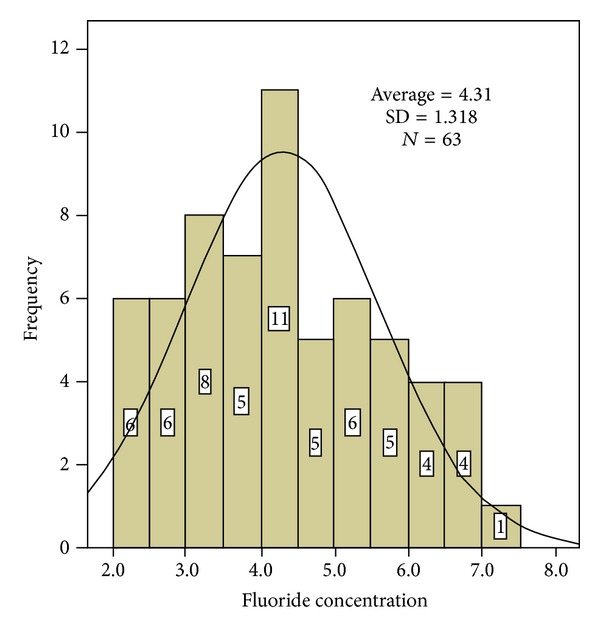
Fluoride concentration distribution in the city of Durango.

**Figure 5:**
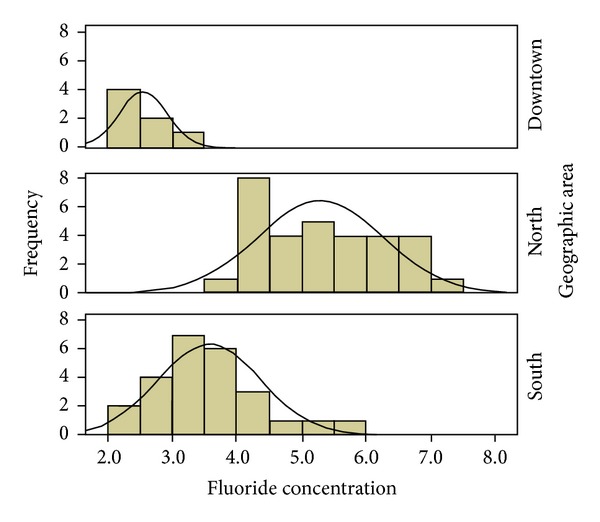
Fluoride concentration frequency distribution in the three regions of the city of Durango.

Figure 4 shows the fluoride concentration frequency distribution for the 63 wells studied. The frequency of fluoride was greater in 11 wells, 27 wells had concentrations between 2 and 4 ppm, and 36 wells had concentrations >4 ppm and Figure 5 shows the fluoride concentration distribution in the three regions studied; the highest fluoride levels were in the northern region, and the lowest levels were in the central region.

## 4. Discussion

The state of Durango is in a rocky volcanic region, and its water is supplied from underground sources. The drinking water fluoride content depends on such rocks' fluoride content and the temperature, pH, and time that the groundwater is in underground aquifers. Minerals, such as clay, that can exchange ions favor fluoride ion diffusion and transport into groundwater [17].

The predominant rocks in the city of Durango are acidic, and experiments showed that the fluoride quantity dissolved from acidic rocks was nearly twice that of basic rocks. Acidic rocks have higher silica concentrations, and when such rocks crystalize, they yield quartz, which indicates a relationship between the groundwater fluoride concentration and surrounding geological formations. Herein, drinking water in the city of Durango had fluoride concentrations greater than 2 ppm, which indicates toxicity and is a risk factor for dental and skeletal fluorosis [18].

The highest concentrations were in the northern region, with fluoride levels up to 7.2 ppm; this region is a densely populated area with a high percentage of the city's preschool-aged children, whose dental enamel and bones are most susceptible to fluoride toxicity [19].

Since 1991, iodized and fluoridated salt has been used in Mexico to prevent dental caries; this policy was implemented taking into consideration that many states have high fluoride concentrations, and it specifies that fluoridated salt should not be distributed in such states. However, despite this recommendation, the work herein identified Durango retailers that sell fluoridated salt [2, 11].

In a study from over a decade ago, fluoride concentrations below 1.5 ppm were observed in the water supply of the city of Durango, while the lowest concentration found in the present study was 2.22 ppm. This discrepancy may be due to the scarcity of water in the state, which led to groundwater extraction from wells as deep as 250 meters, which is an aquifer depth with higher fluoride levels [20]. In the center of the city of Durango, where middle-class families primarily reside, the fluoride concentrations in drinking water samples did not exceed 3.31 pm. However, in the outlying regions, where lower-income families primarily reside, the fluoride concentrations were as high as 7 ppm, which suggests that the populations exposed to the highest levels of fluoride toxicity are the city's most disadvantaged residents [21].

Excessive fluoride exposure should be considered a serious public health problem in the state of Durango. Fluoride toxicity affects children more severely than adults because the compound accumulates more rapidly in growing and developing children's bones and manifests as genu varum or bowleggedness [22].

Dental fluorosis is caused by fluoride ingestion during odontogenesis. The severity of this condition is related to the fluoride concentration ingested, exposure period duration, and individual susceptibility, which may depend on risk factors such as calcium deficiency, malnutrition, and kidney disorders that affect the body's acid-base equilibrium [23].

In acidosis, the urine production rate decreases, while in alkalosis, this rate increases. Animal studies have demonstrated that acidosis adversely affects tooth enamel formation. Other studies report that populations at altitudes greater than 1500 meters above sea level retain fluoride in their tissues and, thus, develop fluorosis at greater rates [24]. Studies have also demonstrated liver damage from overexposure to fluoride sources, with effects that include protein synthesis inhibition, primarily due to peptide chain disruption, and this interference with peptide chain formation in ribosomes has caused reduced serum levels of total proteins and albumen to be reported [24].

The study herein was conducted under time constraints. Because the water samples were collected over nine months (in a period of drought), it is recommended that, in future studies, samples are collected over longer time periods to identify seasonal variations. Determining the fluoride concentrations in Durango is important because disseminating such results may sensitize the public health sector, particularly stakeholders with an interest in water quality, such as pediatricians, who play a fundamental role because the primary fluoride susceptibility period is the first years of life. During this period, children have more contact with pediatricians than with dentists.

Based on the work herein, it is important to establish purification systems to eliminate toxic substances from drinking water or seek alternative drinking water sources. From the first stages of infancy, children's exposure to fluoride should be controlled by avoiding fluoridated salt and limiting other fluoridated products, such as toothpaste, which are an additional source of fluoride exposure; such measures are particularly important for children under six years of age [25].

Furthermore, systematically monitoring of chemical water quality is critical for the different wells and household water sources during rainy periods and droughts. It is also important to perform radiological or densitometric studies on adult inhabitants of the regions to determine significant alterations in bone density because prolonged and excessive fluoride ingestion can increase the risk of bone fractures in adults due to skeletal fluorosis. Furthermore, we recommend that sanitary authorities educate the population on preventive measures and implement specific programs to improve the quality of life in the city of Durango.

## Figures and Tables

**Table 1:** Mean fluoride concentrations by region in the city of Durango. The three geographic regions yielded significantly different values using ANOVA (*P* < 0.00).

Geographic region	Mean fluoride concentration (ppm)	Number of wells	Standard error	Confidence interval (95%)
Central region	2.551	7	0.137	[2.820, 2.283]
Northern region	5.300	31	0.170	[5.633, 4.968]
Southern region	3.582	25	0.157	[3.890, 3.274]
